# Advances in Anti-aging Procedures: A Comprehensive Review of Surgical and Non-surgical Rejuvenation Techniques

**DOI:** 10.7759/cureus.92666

**Published:** 2025-09-18

**Authors:** Yasir Rashid, Cielo Estela-Fernandez, Azkah Sardar, Denyse Deowan, Omara M Safdar, Iqrah A Issimdar, Sergio Camilo Torres Céspedes, Nabia Dawood, Ramsha Ali, Alisson B Silva

**Affiliations:** 1 Dermatology, Al-Mustansiriyah University, Baghdad, IRQ; 2 Dermatology, Universidad Científica del Sur, Lima, PER; 3 Dermatology, Liverpool School of Tropical Medicine, Liverpool, GBR; 4 Dermatology, University of Glasgow, Glasgow, GBR; 5 Medicine and Surgery, San Fernando Teaching Hospital, San Fernando, TTO; 6 Dermatology, Fatima Jinnah Medical University, Lahore, PAK; 7 Health and Agriculture, University College Dublin, Dublin, IRL; 8 General Practice, Universidad Nacional de Colombia, Bogotá, COL; 9 Dermatology, Punjab Medical School, Faisalabad, PAK; 10 Medicine and Surgery, Peoples University of Medical and Health Sciences, Hyderabad, PAK; 11 General Surgery, UNIME School of Medicine, Salvador, BRA

**Keywords:** aesthetic medicine, botulinum toxin, dermal fillers, innovation in cosmetology, laser therapy, minimally invasive procedures, skin boosters

## Abstract

Advances in medical science have led to longer lifespans, increasing global demand for cosmetic procedures aimed at preserving or enhancing youthfulness. A wide range of clinical options now targets facial ageing, offering invasive and non-invasive treatments tailored to individual needs, medical factors, and financial considerations. With recent advances in aesthetic technologies and emerging evidence-based results, this narrative review explores recent innovations in anti-ageing interventions, focusing on their effectiveness and potential side effects.

This narrative review aims to comprehensively evaluate anti-ageing procedures, including both surgical and non-surgical techniques. It seeks to synthesize current evidence to assess the safety, efficacy, and impact of these interventions on patient outcomes.

A narrative review was conducted by searching PubMed, MEDLINE, Scopus, and Web of Science for articles published between 2010 and 2025. Keywords included "anti-ageing," "facial rejuvenation," "cosmetic procedures," and "aesthetic treatments." Eligible sources were peer-reviewed original research, systematic reviews, meta-analyses, and clinical guidelines. Non-English articles, conference abstracts without full texts, and studies unrelated to facial treatments were excluded. Formal quality assessment and statistical analysis were not performed.

Of 640 articles identified, 144 were included in the final synthesis. Findings highlight the multifactorial nature of facial ageing and recent advances supporting personalized interventions to enhance patient outcomes and quality of life.

## Introduction and background

The global anti-ageing industry has experienced substantial growth, driven by rising demand for procedures that maintain or restore a youthful appearance. Historically, societies have associated certain physical traits with beauty, youth, and health. While beauty standards have evolved, youthful characteristics remain consistently valued [1]. Advances in medicine and increased life expectancy have further fueled the desire for both surgical and non-surgical cosmetic interventions. Between 2010 and 2023, aesthetic procedures grew by 3.4% globally, with 34.9 million treatments performed in 2023 alone [2]. Interestingly, the most recent statistics for 2024, when compared to 2023, showed an overall 4.8% decrease in all aesthetic/cosmetic procedures. Driven primarily by surgical interventions with a reduction of 6.7%, compared to non-surgical ones of 3.1% [3].

A wide range of procedures now targets facial aging, offering invasive and non-invasive options tailored to individual preferences, medical considerations, and financial means. Patients increasingly seek a “natural look,” favoring subtle enhancements that preserve authenticity [4]. Non-invasive options, such as injectables (botulinum toxin, dermal fillers) and energy-based treatments (laser resurfacing, radiofrequency), provide reversible or short-term results compared with surgical approaches [5]. Management has become more comprehensive, guided by evolving regulations and a focus on holistic, patient-centered research [2].

This narrative review provides a comprehensive overview of anti-ageing interventions, examining both surgical and non-surgical techniques, their efficacy, and potential side effects [5-6]. It evaluates common complications associated with procedures such as botulinum toxin, platelet-rich plasma (PRP), and dermal fillers, including hyaluronic acid (HA), as well as their effects on specific facial regions, such as the lips, jawline, and periorbital area [7]. Emerging techniques, including high-intensity focused ultrasound (HIFU) and PRP, show promise for eyelid and lip rejuvenation but require further research in dermatological contexts. Potential adverse effects include vascular occlusion from fillers and ptosis or muscle weakness following botulinum toxin, highlighting the importance of evidence-based guidelines and robust complication management strategies [6].

Although both surgical and non-surgical approaches are generally safe and effective, continuous research is essential to refine protocols, optimize patient outcomes, and enhance safety. This review consolidates current knowledge on anti-ageing interventions, providing clinicians with an evidence-based framework to guide practice.

## Review

Materials and methods

A narrative review was conducted to summarize current evidence on surgical and non-surgical anti-aging procedures. Electronic databases, including PubMed, MEDLINE, Scopus, and Web of Science, were searched for articles published between 2010 and 2025. The search strategy used the following keywords: ("anti-aging procedures" OR "rejuvenation techniques" OR "facial rejuvenation") AND ("surgical techniques" OR "surgical rejuvenation" OR "facelift surgery" OR "blepharoplasty") OR ("non-surgical treatments" OR "non-invasive rejuvenation" OR "Botox" OR "dermal fillers" OR "laser skin resurfacing" OR "radiofrequency skin tightening" OR "PRP therapy" OR "HIFU").

Eligible sources included peer-reviewed original research, systematic reviews, meta-analyses, and clinical guidelines focused on facial anti-aging interventions. Non-English articles, conference abstracts without full texts, and studies unrelated to facial treatments were excluded. Findings were synthesized thematically to explore recent advances, effectiveness, and safety profiles of available treatments. As a narrative review, formal quality assessment and statistical analysis were not performed.

Inclusion and exclusion criteria were applied to ensure methodological rigor and relevance. Studies published within the past five years that investigated advances in anti-aging procedures, encompassing both surgical and non-surgical rejuvenation techniques with adult participants (≥18 years) were included. Studies were excluded if they were published in languages other than English, involved pediatric or pregnant populations, or were conducted on animal models.

Results

The initial review yielded 640 papers. After applying inclusion and exclusion criteria, 163 studies were selected for further review. Following abstract screening, 144 studies met the full inclusion criteria and were included in the analysis.

This review provides valuable insights for both clinicians and patients into how surgical and non-surgical procedures influence facial aesthetic outcomes and skin rejuvenation strategies. Both types of interventions were found to significantly improve quality of life, with many patients reporting high satisfaction with results [8]. In contemporary culture, preserving a youthful and pleasant facial appearance is increasingly linked to emotional well-being and self-esteem [9].

**Figure 1:**
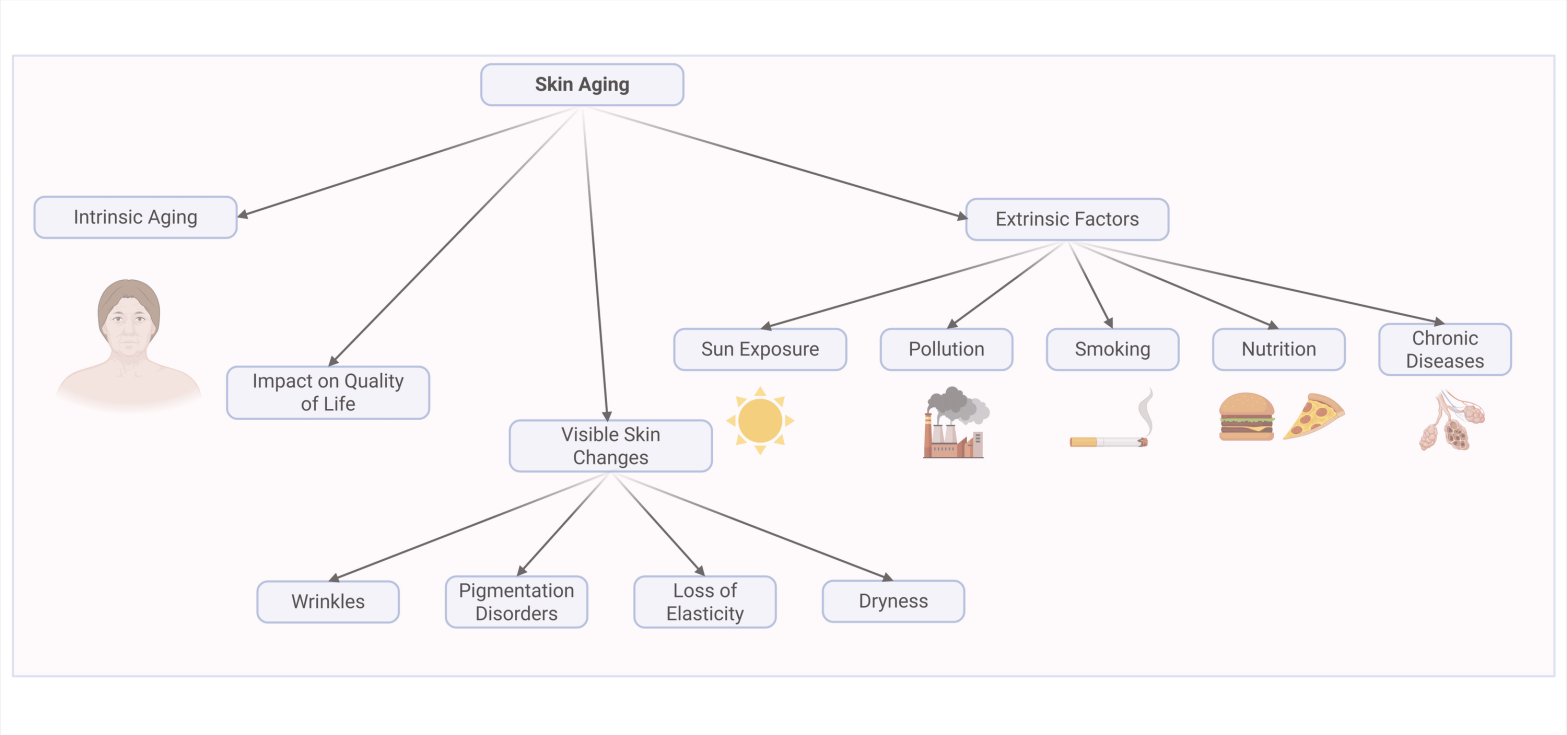
Conceptual map of skin aging Skin aging involves a multifactorial scope of factors and results from the interplay between them. Intrinsic factors, such as chronological and genetic aging and extrinsic factors like sun exposure, pollution and smoking, contribute to visible skin changes which impact on individual's quality of life [10,11,12,13,14] Image credits: Cielo Estela-Fernandez, Sergio Camilo-Torres; created in BioRender.com

Skin aging is a multifactorial process driven by intrinsic mechanisms (including reduced proliferative capacity, genetic predisposition, and hormonal changes) and extrinsic factors such as chronic UV radiation, pollution, and lifestyle choices (diet, smoking, alcohol, sleep, and skincare practices). They collectively contribute to the phenotypic manifestations of aging, presenting as wrinkles, sagging, and hyperpigmentation. The structure and function vary by location, sex, and ethnicity, reflecting the complexity of this process. At the cellular level, complex molecular pathways related to oxidative stress, collagen metabolism, melanogenesis, and dermal integrity play a role [10-12]. The accumulation of senescent cells disrupts the clearance mechanisms of macrophages (they inhibit the secretory phenotype associated with senescence), favoring further senescent cell buildup. Moreover, the secretion of proteins associated with skin aging has systemic implications, accelerating whole-body aging and impairing the function of other organs [13-14]. A conceptual map of skin ageing is presented in Figure 1 to illustrate these interrelated factors.

Injectables (Botulinum Toxin and Dermal Fillers)

Injectables, particularly botulinum toxin and dermal fillers (such as hyaluronic acid (HA), calcium hydroxyapatite (CaHA), poly-L-lactic acid (PLLA)), remain foundational in non-surgical rejuvenation. Botulinum toxin showed consistent short-term improvements in dynamic wrinkles and expression lines, with experts noting its role in managing facial pore size and treating masseter hypertrophy and gummy smile [15-18].

Moreover, various filler materials such as PLLA, polycaprolactone (PCL), and polymethylmethacrylate (PMMA) in hydrogel form have gained attention due to their effects in relation to volume gain, collagen stimulation (bioactive matrices), and patient satisfaction. However, its safety and effectiveness require further investigation [19]. In contrast, the combined use of HA with CaHA offers promising results in facial rejuvenation, providing both volumization and long-term collagen stimulation, with good tolerability, a high satisfaction rate, and minimal adverse effects such as pain, edema, nodules, and inflammation [20].

Interestingly, the synergistic use of botulinum toxin with dermal fillers seems to optimize the results with greater patient satisfaction in the reduction of dynamic wrinkles and masseteric hypertrophy. In addition, when combined with Profhilo gel, there's evidence of better and safer effects in neck rejuvenation. Nevertheless, caution should be taken with pre-existing conditions such as weakness, instability, or curvature due to the risk of exacerbation. Despite its benefits, this approach is temporary (3-4 months), carries a risk of asymmetric results, ecchymosis, ptosis, or headache, and is expensive. In rare cases, dysphagia, facial paralysis, botulism, and death have been reported. This highlights the importance of expanding research seeking to refine techniques and improve patient safety [21].

The rationale for HA-based fillers is supported by the fact that aging implies lower endogenous production of HA. Consequently, HA fillers aim to restore the natural loss of moisture and volume. They optimize facial contours without surgery, minimal downtime, and relatively low risks. Still, studies of long-term effects and patient satisfaction are needed [22]. Recent evidence also suggests that HA fillers were associated with improvements in skin radiance and hydration, probably due to their role in optimizing the extracellular matrix, collagen stimulation, and anti-inflammatory effects. No changes in elasticity or pigmentation were evident [23].

When selecting a filler, clinicians must consider the rheological properties, the tissue depth, and patient-specific anatomic variations. HA fillers have certain advantages; their reversibility makes them suitable for less experienced practitioners, whereas non-HA fillers may be an option for longer-lasting effects despite the risks. Distinguishing between biphasic HA fillers, which offer strong volumization with difficult molding, and monophasic fillers, which have greater cohesiveness and softness, as well as the analysis of viscoelastic parameters, allows more precise results and treatment individualization [24].

From a clinical perspective, HA fillers have demonstrated significant efficacy in midface volumization, tear trough correction, and lip augmentation. The skin boosters based on HA also improve skin hydration and elasticity, aligning with modern demands for natural-looking enhancements [23, 25-27]. However, complications such as vascular occlusion, visual loss, and immune reactions were emphasized, requiring careful patient selection and anatomical knowledge [28-29].

Currently, HA fillers are the most widely used, especially in the correction of nasolabial folds. They are considered safer, and hyaluronidase can provide timely management of their associated complications. Self-limiting adverse effects such as pain, erythema, edema, and nodules may frequently appear. On the other hand, there are moderate to severe complications such as infection, filler migration, and vascular occlusion. Outcomes depend on multiple variables, including practitioner experience, material properties, injection techniques, and patient-specific factors [30]. Moreover, in relation to the management of acne scars, a dual-plane technique is described that acts on the dermal and subdermal components of the scar using HA [31].

Finally, in an effort to facilitate clinical practice, a consensus of experts has proposed a framework that simplifies the Restylane portfolio of HA fillers, providing guidance according to facial regions, exceptions, and techniques and seeking to unify recommendations, improve reproducibility, and optimize decision-making [32].

Bio-Stimulatory and Regenerative Therapies

Bio-stimulatory fillers such as CaHA, PLLA, and PMMA provide longer-lasting results by stimulating collagen synthesis. CaHA has a dual role in providing immediate contouring and delayed regenerative effects [33]. 

Biostimulators, including PLLA, CaHA, platelet-rich plasma (PRP), and platelet-rich fibrin (PRF), have shown beneficial effects in local rejuvenation by increasing collagen production. Their efficacy depends on the subtype, delivery method, and adjuvant therapies. However, available data remains heterogeneous, with no clear consensus regarding preparation, administration, and measurement of results. This highlights the need for further validation [34].

In the field of facial rejuvenation, PLLA has emerged as a safe (comparable to HA and collagen fillers) and effective option, improving skin quality and elasticity with high reported satisfaction from patients. In order to minimize adverse effects, dilution protocols, hydration time, and various administration methods have been reported. Nonetheless, studies are heterogeneous, which underscores the need for long-term studies to evaluate their safety and effectiveness, as well as the study of new formulations [35].

CaHA also has specific applications in hand rejuvenation, where the novel dilution/hyperdilution technique has shown good results [36]. Moreover, the combinations of biostimulators with botulinum toxin, dermal fillers, and energy-based devices appear to provide synergistic improvements in skin laxity and wrinkle reduction. On a cellular level, effects on enhanced fibroblastic activity have been proposed: CaHA through TGF-B and macrophage activation, PLLA through the degradation of lactic acid and HA fillers via CD44-MAPK signaling and stimulation by hydration. These processes are further enhanced by the control of thermal and mechanical damage mediated by energy-based modalities such as high-intensity microfocused ultrasound (HIFU), radiofrequency (RF) microneedling, and intense pulsed light (IPL). Additionally, botulinum toxin A contributes by reducing dermal tension and stabilizing collagen synthesis. Despite these promising combinations, robust research is necessary to validate their effectiveness, standardize protocols, and establish long-term safety [37].

While side effects such as erythema and localized swelling are typically transient, experts caution that granulomatous reactions and nodule formation with biostimulants, though rare, require standardized treatment protocols [7, 38, 39].

On the other hand, polynucleotides (PN) and PRP have gained attention for their ability to improve elasticity, tone, and reduce fine wrinkles, especially around delicate areas like the eyelids [5, 40].

PNs are noteworthy as natural tools that stimulate growth, tissue repair, hydration, collagen production, and overall skin quality with long-term effects. Their safety profile appears to be better than HA fillers or collagen-stimulating agents. Compared to PRP or PRF, they also offer advantages in terms of lower costs, reduced procedure time, and less operator dependence [41].

Similarly, PRP therapy has shown promising outcomes in the field of women with androgenetic alopecia, telogen effluvium, and female pattern hair loss. It exerts its effects through growth factors such as PDGF and VEGF that stimulate stem cells and angiogenesis, being a natural element with a favorable safety profile. Nevertheless, it is important to highlight the heterogeneity of the techniques and external influencers, and the need to determine their effectiveness, durability, and synergistic properties with long-term studies [42]. Among preparation methods, the single-spin centrifugation technique has shown superior outcomes [43].

Kybella (deoxycholic acid) is an FDA-approved adipocytolytic agent for nonsurgical lipolysis. Subcutaneous injections induce adipocyte necrosis, which has been reported to improve up to 70% in submental contour [9]. Common adverse effects include bruising, alopecia, and numbness [9]. Although surgical and laser-assisted liposuction remain the gold standard for subcutaneous fat reduction, injection lipolysis offers advantages such as minimal downtime, reduced pain, and less invasiveness, but requires careful patient selection, proper technique, and multiple sessions for optimal outcomes [9].

Energy-Based Devices

Lasers (ablative, non-ablative, IPL, erbium-doped yttrium aluminium garnet (Er-YAG), Q-switch, Pico) have demonstrated efficacy in the treatment of photodamaged skin, fine lines and wrinkles, pigmentation, tone and texture improvement, short recovery time, session tailored to individual needs, and conform to irregular surfaces. It is confirmed that it could be used as a utility in treating periorbital wrinkles and photodamaged skin [44-45]. Requires multiple treatment sessions, yields gradual results, and necessitates limiting sun exposure to reduce the risk of hyperpigmentation [44-45].

LED light therapy (particularly at wavelengths of 633 nm, 830 nm) treats aging skin, wrinkles, and acne by stimulating collagen and elastin, but is contraindicated in patients on photosensitizing drugs or with skin cancer or inherited eye disease [46].

RF technologies (mono-, bi-, tri-, multipolar, or combined) enhance hydration, reduce sun damage, and minimize aging signs by stimulating collagen and elastin synthesis are used for acne, scarring, skin rejuvenation, tightening, and stretch marks [9].

Jointly, RF and radiofrequency microneedling (RFM) are reported as tools of moderate effectiveness and adequate safety profile in the management of rhytides, hyperpigmentation, and scar management. However, further studies are required regarding its differential effect and effectiveness across different skin phototypes and populations [47].

Ultrasound-based therapies, including HIFU and Ultherapy, provide deeper stimulation to the SMAS layer, promoting lifting and tightening with minimal downtime. It has proven efficacious in the treatment of periocular rhytids, brow ptosis, and mild to moderate skin laxity. Experts acknowledged mild pain and temporary erythema as manageable adverse effects, making these methods ideal for patients seeking non-invasive alternatives to surgery [48].

HIFU has been reported as a minimally invasive tool, with positive semi-permanent effects on facial laxity, nasolabial fold, jawline, and neck (up to six months). It exerts its effect through focal and selective necrosis of adipose cells and collagen remodeling. The safety profile is good; it is associated with transient events such as erythema, pain, edema, or ecchymosis. Although rare, there are reports of neuromuscular dysfunction, scarring, or bleeding. It is an expensive option, which requires strengthening the evidence of its results [49]. Its effectiveness is influenced by energy levels, planes to be treated, and focal depths [50]. HIFU therapy applied for sagging skin has been associated with short- to long-term improvements in skin laxity. With a low rate of adverse effects. Nevertheless, available evidence related to its safety and efficacy remains limited, and doesn't suggest superiority compared to other similar technologies [51].

Combination approaches, such as microfocused ultrasound with visualization (MFU-V) and calcium hydroxyapatite with carboxymethylcellulose (CaHA-CMC), have been associated with collagen neoformation and elastin synthesis, optimizing the quality of the skin with an optimal safety profile. Its evidence is limited and requires further studies to validate its long-term effectiveness and standardize use protocols [52].

Mechanical Lifting (Thread Lifting)

Thread lifting with PLLA, PCL, or polydioxanone (PDO) threads provides a minimally invasive solution for addressing surface irregularities, fine lines, and volume loss. In addition, it improves skin texture and tightness, offering clinicians greater control over tissue positioning [18]. 

PDO threads are considered a promising minimally invasive modality of facial rejuvenation. However, there is limited data regarding outcomes across different populations, skin prototypes, and safety and efficacy parameters regarding its different types (spiculated, monofilament, PDO, PLLA, or PCL) [53].

Complications associated with threads depend on the material, fixation, texture, and location. Regarding the material, PCL has been associated with greater sensitivity and ecchymosis, while PDO showed less inflammation but a higher infection rate, and PLLA showed an intermediate profile. In terms of fixation and texture, barbed threads (frequently PDO) have been linked with greater erythema, ecchymosis, sensitivity, and infection compared to smooth designs. Smooth threads, due to their higher mobility, are often related to inflammation, erythema, and ecchymosis. Finally, localization also influences the risk profile: midface placement (with thinner skin and abundant vasculature) presented greater inflammation, ecchymosis, and sensitivity, while its use in the jawline (close to trigeminal branches and high glandular density) predisposed to paresthesia and infection [54]. All these complications underline the need for practitioner expertise and post-treatment monitoring [39].

Chemical peels and exfoliants: Chemical peels remain a cost-effective option for superficial rejuvenation, effectively targeting acne scars, melasma, and mild photoaging. They are commonly used as adjunctive treatments within broader aesthetic regimens [55], [56]. Side effects like post-inflammatory hyperpigmentation and irritation were reported, especially in darker skin tones or when used without sun protection or medical supervision [39].

Microneedling and Combination Modalities

Traditional microneedling, radiofrequency microneedling, and nano-needling demonstrated marked improvements in skin quality by promoting collagen production and neovascularization. These techniques are particularly effective in scar treatment and skin rejuvenation, especially when combined with PRP or vitamin-enriched serums. They are generally well-tolerated, with minor erythema and edema subsiding within 48-72 hours [39].

Fractional radiofrequency microneedling (FRM) has shown comparable efficacy to fractional lasers, including CO2, Er:Glass, and Er:YAG, with the advantage of greater tolerability, shorter execution time, and fewer adverse effects (mainly self-limited, such as post-inflammatory hyperpigmentation). Despite these interesting findings, more robust studies are needed to establish long-term efficacy, define effects on different prototypes, and optimize treatment parameters [57].

In the context of acne scars, microneedling techniques have shown greater effectiveness mainly in combination with chemical peels, but also with HA, botulinum toxin A, PRP, and laser therapy [58]. On the other hand, this technique, combined with minoxidil, has been associated with improved outcomes in patients with androgenetic alopecia. Interestingly, no significant changes were found in relation to differences in depth, duration of treatment, or technique (device used) [59].

Expert Perspective

Researchers across the reviewed literature consistently emphasize that personalized, multimodal approaches yield superior outcomes compared to monotherapies. The anatomical depth of action should guide technique selection to optimize outcomes and minimize complications [60]. Overall, non-surgical interventions continue to grow in popularity due to their effectiveness, convenience, and lower risk profile-especially for younger populations seeking preventive or subtle enhancements.

For ease of comparison, a summary of the main non-surgical anti-ageing techniques, including their mechanisms and clinical outcomes, is provided in Table 1:

**Table 1:** Comparative study of non-surgical anti-aging techniques IPL, intense pulsed light; Er-YAG, erbium-doped yttrium aluminium garnet; HA, hyaluronic acid; CaHA, calcium hydroxylapatite; PLLA, poly-l-lactic acid; PMMA, polymethyl methacrylate; PN, polynucleotides; PDRN, polydeoxyribonucleotide; PDO, polydioxanone; PCL, polycaprolactone; TCA, trichloroacetic acid; AHAs, alpha-hydroxy acids; BHAs, beta-hydroxy acids; PRP, platelet-rich plasma; RF, radiofrequency

Category	Technique	Indications	Advantages	Limitations	Key references
Energy-based devices	Laser (ablative, non-ablative, IPL, Er-YAG, Q-switch, Pico)	Photodamaged skin, fine lines and wrinkles, pigmentation, tone, and texture improvement	Short recovery time, session tailored to individual needs, conforms to irregular surfaces	Requires multiple sessions, subtle results, and sun exposure should be limited to avoid hyperpigmentation	Heidari Beigvand et al., 2020 [44]; Machado et al., 2021 [45]
	LED light therapy (633 nm, 830 nm)	Facial aging, fine lines and wrinkles, active acne vulgaris	Photo-modulation leads to wrinkle reduction, improved elasticity, collagen, and elastin synthesis	Not suitable for individuals on photosensitizing medications or with a history of skin cancer or inherited eye disease	Lee et al., 2024 [46]
	Radiofrequency (mono, bi, tri, multipolar, combination)	Acne, acne scars, general scarring, skin rejuvenation, tightening, stretch marks	Improves hydration, sun damage, and signs of aging by synthesizing collagen and elastin	-	Manuskiatti et al., 2025 [9]
	Ultrasound therapy (ultherapy)	Periocular rhytids, brow ptosis, mild to moderate skin laxity, and infraorbital hollowing	Minimally invasive, less pain, no downtime	Pain, bruising, transient paralysis, edema, erythema, numbness, dyspigmentation	Tao et al., 2025 [48]
Injections	Botulinum toxin (onabotulinumtoxin A, abobotulinumtoxin A, incobotulinumtoxin A, daxibotulinumtoxin A, rimabotulinumtoxin A)	Dynamic wrinkles, bulky muscles, smoothing enlarged pores, androgenic alopecia, and gummy smile	No surgery needed, immediate results, economically accessible, and less time-consuming	Side effects include pain, swelling, bruising, hematoma, hypertonia, and swallowing and breathing difficulties	Camargo et al., 2021 [15]; Vachiramon et al., 2025 [16]; Fatani et al., 2022 [17]; El Hawa et al., 2024 [39]; Santis et al., 2025 [61]
	HA (cross-linked and non-cross-linked)	Tear trough, midface volumization, lower face rejuvenation, skin hydration, lip augmentation	BDDE improves the durability of the filler	Immune response manifests in various ways, such as vascular occlusion and visual loss	Hong et al., 2024 [18]; Zhou et al., 2024 [23]; Woodward et al., 2023 [25]; Trinhet al., 2022 [26]; Wongprasert et al., 2022 [27]; Soares et al., 2022 [28]; Kato et al., 2022 [29]; El Hawa et al., 2024 [39]; Scardua et al., 2024 [62]; Go et al., 2023 [63]
	Bio-stimulation fillers (CaHa, PLLA, PMMA)	Facial contouring, striae cutis destensae, facial soft tissue augmentation, correction of fat loss and nasolabial fold, thread lifting, increasing volume	Long-lasting results, environmentally friendly, minimal immune response	Visual loss, granulomatous foreign body reaction, migration of the filler material, chronic inflammation, swelling, formation of nodules, persistent skin discoloration	Christen et al., 2022 [7]; Aguilera et al., 2023 [33]; Alsharif et al., 2023 [38]; El Hawa et al., 2024 [39]; Scardua et al., 2024 [62]; Go et al., 2023 [63]
	Skin booster (HA)	Skin hydration assists skin aging	-	-	Yi et al., 2024 [5]; Zhou et al., 2024 [23]
	PRP	Induction of regeneration and anti-inflammatory effect	Heals burns, acne scar correction	Local inflammation, transient edema	Manole et al., 2024 [40]
	Polynucleotides (PN, PDNR)	Improves pore size, tone, melanin levels, wrinkles, and sagging	-	Facial erythema	Yi et al., 2024 [5]
	Mesotherapy cocktails (amino acid mix, HA, vitamins C and E)	No data	Increase hydration, no downtime	Erythema, ecchymosis, edema, infections	El Hawa et al., 2024 [39]
	Stem cells and exosomes	No data	Promotes cellular cohesion, increases elastin production no data	-	Yi et al., 2024 [5]
Other non-invasive therapies	Electrical stimulation (high facial electrical stimulation)	Skin laxity, muscle toning, improves wrinkles, reduces pore volume, decreases pigmentation	Non-invasive, both the skin and the underlying musculoaponeurotic framework are targeted to effectively enhance results	-	Manuskiatti et al., 2025 [9]
Mechanical lifting	Threading lifting (PDO, PLLA, PCL)	Reinforce dermal tissue, fine lines, tear trough deformities, surface irregularities, and volume restoration	Increase volume, improve skin texture and tightness in treated areas, greater control over the amount injected, and reduced risk of hematoma	Swelling tissue-based inflammation	Hong et al., 2025 [18]; El Hawa et al., 2024 [39]
Chemical exfoliations and peels	Chemical peels (superficial, medium, and deep peels: TCA, AHAs, BHAs, mandelic, phenol/ modified phenol, kojic acid, enzymatic peels (natural enzymes from papaya and pineapple)	As an adjunctive therapy management of rhytids and skin hydration, firmness, tonicity, and elasticity. Acne scars, melasma, and hyperpigmentation	Non-invasive, simple, cost-effective, and time-efficient; improve mild postinflammatory hyperpigmentation, superficial/ mixed melasma, and photoaging signs	Transient erythema, irritation, and burning. Hypertrophic scarring inflammation, post-inflammatory hyperpigmentation, contraindicated in pregnancy, breastfeeding, history of keloid, active infection, and immunosuppressive therapy	El Hawa et al., 2024 [39]; Bravo et al., 2022 [55]; Pathak et al., 2020 [56]
Microneedling	Traditional microneedling (dermaroller, electronic pen)	Treatment of facial wrinkles, acne, and burn scars, enlarged pores, melasma, androgenetic alopecia, and alopecia areata	Stimulate scarless wound healing, minimal epidermal damage, promote collagen production, neovascularization, and skin regeneration	Contraindication for skin-sensitive or healing disorders. Mild redness, swelling, and peeling with 2-3 days of downtime	El Hawa et al., 2024 [39]
	RF microneedling (fractional microneedling RF)	Delivery of radiofrequency, HA, vitamins, or PRP	Moderate efficacy	-	Li et al., 2024 [58]
Fat reduction and skin tightening	Kybella (deoxycholic acid )	Localized fat deposition	-	-	Shome et al., 2023 [8]
	RF-based tightening (Thermage, Exilis)	Stimulate collagen production, tone facial muscles	Synergistic lifting and tightening effect	Pain, short duration of results	Manuskiatti et al., 2025 [9]

Discussion

Emerging research on skin aging reveals that it stems from both intrinsic chronological factors and cumulative exposure to various external influences [16-48]. Multiple strategies now exist to slow down and reverse the signs of aging. This narrative review showcases the increased complexity and variety of surgical and nonsurgical techniques targeting the multimodal approach to facial ageing.

Minimally invasive, non-surgical options-such as lasers, radiofrequency, ultrasound (e.g., HIFU, chemical peels, and injectables (e.g., Botox and dermal fillers)-are becoming more popular [17,55,61]. Many people are drawn to these treatments because they are generally safer, more convenient, and involve less downtime than surgery. Injectables remain a mainstream form of nonsurgical facial rejuvenation. Botulinum toxin is highly effective for dynamic wrinkles [18], and it is validated in a Cochrane review [15], while dermal fillers (especially HA and biostimulatory types like CaHA or PLLA) restore lost volume and improve skin texture [7, 33, 38, 62]. The tear trough correction and midface volumetric augmentation were documented in various studies by using the dermal fillers [25-27]. More novel forms of injectables, such as skin boosters, PRP, and polynucleotides, are emerging as supportive treatments to enhance hydration and collagen production [40, 63]. They have also been shown to be helpful in eyelid rejuvenation, perioral improvement, and reducing wrinkles around the ocular region. Vascular occlusion, ptosis, and muscle weakening are potential side effects of these rejuvenation methods, particularly when fillers are utilized.

**Figure 2:**
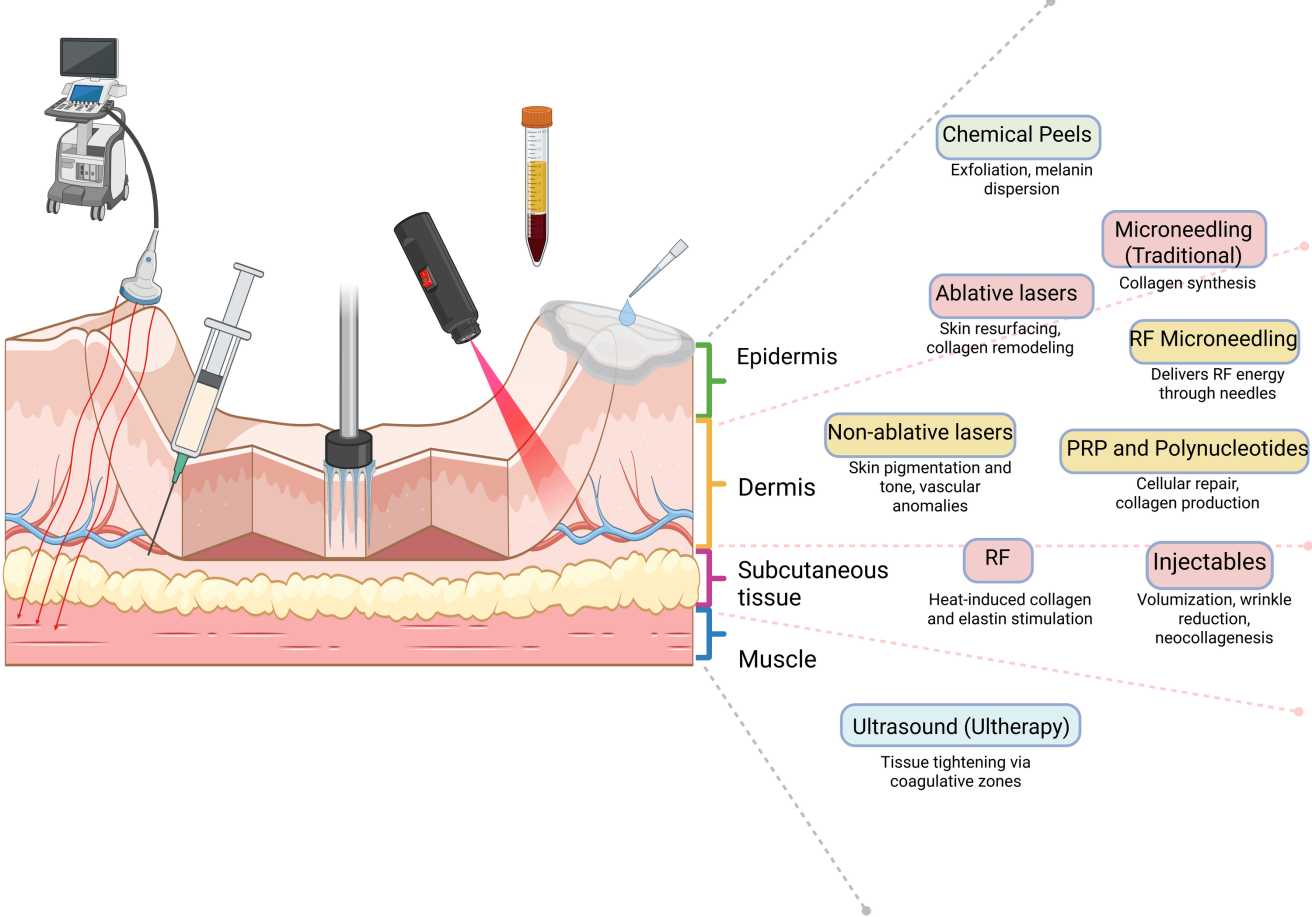
Skin layers and depth of action of anti-aging technologies This diagram shows how various anti-aging technologies work on different skin depths. Chemical peels and microneedling target the epidermis and improve cutaneous renewal and collagen stimulation. Lasers, radiofrequency, PRP, and polynucleotides act within the dermis, enhancing repair, tone, pigmentation, and elasticity. Injectables reach the hypodermis to restore volume and reduce wrinkles, while MFU penetrates deeper, acting on fat layers and the SMAS to promote muscular tightening and lifting. Combining and matching these technologies is key to an effective and personalized treatment [4,5,9,16,39,45]. Image credits: Sergio Camilo-Torres, Cielo Estela-Fernandez; created in BioRender.com RF: radiofrequency; PRP: platelet-rich plasma, MFU: microfocused ultrasound; SMAS: superficial musculoaponeurotic system

Energy-based devices (e.g., lasers, LED therapy, RF, ultrasound, HIFU) effectively improve skin laxity, texture, and pigmentation, especially when tailored to specific skin types and concerns [44]. Chemical peels and microcurrent stimulation are effective for superficial rejuvenation and muscle toning [9, 56]. However, their results are less apparent than those of other interventions. Combination therapies often yield better outcomes than single treatments. They offer synergistic benefits and can address multiple aspects of aging simultaneously. The skin layer and depth of action diagram (Figure 2) help to visualize how various technologies interact with different anatomical targets.

The observations made in this review agree with recent literature, which highlights the reconstructive shifts within aesthetic medicine and the amplification of evidence that validates non-invasive facial rejuvenation techniques.

The incorporation of energy-based technologies has also received considerable attention, where the use of fractional laser therapies results in marked improvement of periorbital rhytids [45], and the synchronized use of radiofrequency with facial electrical stimulation greatly improves skin laxity and elasticity in Asian populations [9]. The chemical peels and microneedling at various depths across skin types for treating conditions such as melasma, post-inflammatory hyperpigmentation, and fine lines [39]. As for bio-stimulatory treatments, there is evidence of CaHA's regenerative properties and immediate contouring effects while noting its long-term collagen production [33]. HA-based skin boosters are proven to enhance hydration and elasticity, which aligns with the contemporary preference for more understated natural augmentations [5, 23].

As noted in the review’s safety assessment, concerns regarding adverse consequences such as vascular occlusion and visual loss must be addressed [28,29]. This adds to the emerging importance of risk minimization in the literature, which is achieved by optimizing technique, anatomical understanding, and careful patient selection.

The demand for aesthetic medicine is growing significantly worldwide, particularly among younger demographics [2]. Thus, there is a pressing need to better understand non-surgical and minimally invasive rejuvenation techniques. This is especially important as patients shift towards less risky treatments with shorter recovery times that can still provide effective aesthetic results [2]. The findings of this research are also of great use to practitioners who can use them to tailor personalized treatment plans for their patients.

Despite offering a comprehensive overview of contemporary anti-ageing interventions, this review is subject to several limitations that merit consideration. Firstly, the differences in study designs, outcomes assessed, and cohorts reviewed in the existing literature pose challenges for direct comparisons. Numerous studies vary in length of follow-up, methods of evaluation (subjective vs. objective skin assessments), and regions of interest, all of which limit the creation of unified clinical guidelines. Additionally, the lack of documentation on practitioner technique, experience, and device calibration (often overlooked factors) can impact efficacy and complication rates. These issues are only superficially addressed in much of the literature reviewed.

Furthermore, certain domains remain understudied, particularly in the dermatological context, regarding microcurrent, electrical stimulation, cryotherapy, and oxygen therapy for aging. Therefore, their findings should not be applied concretely until better and more extensive RCTs are undertaken.

**Figure 3:**
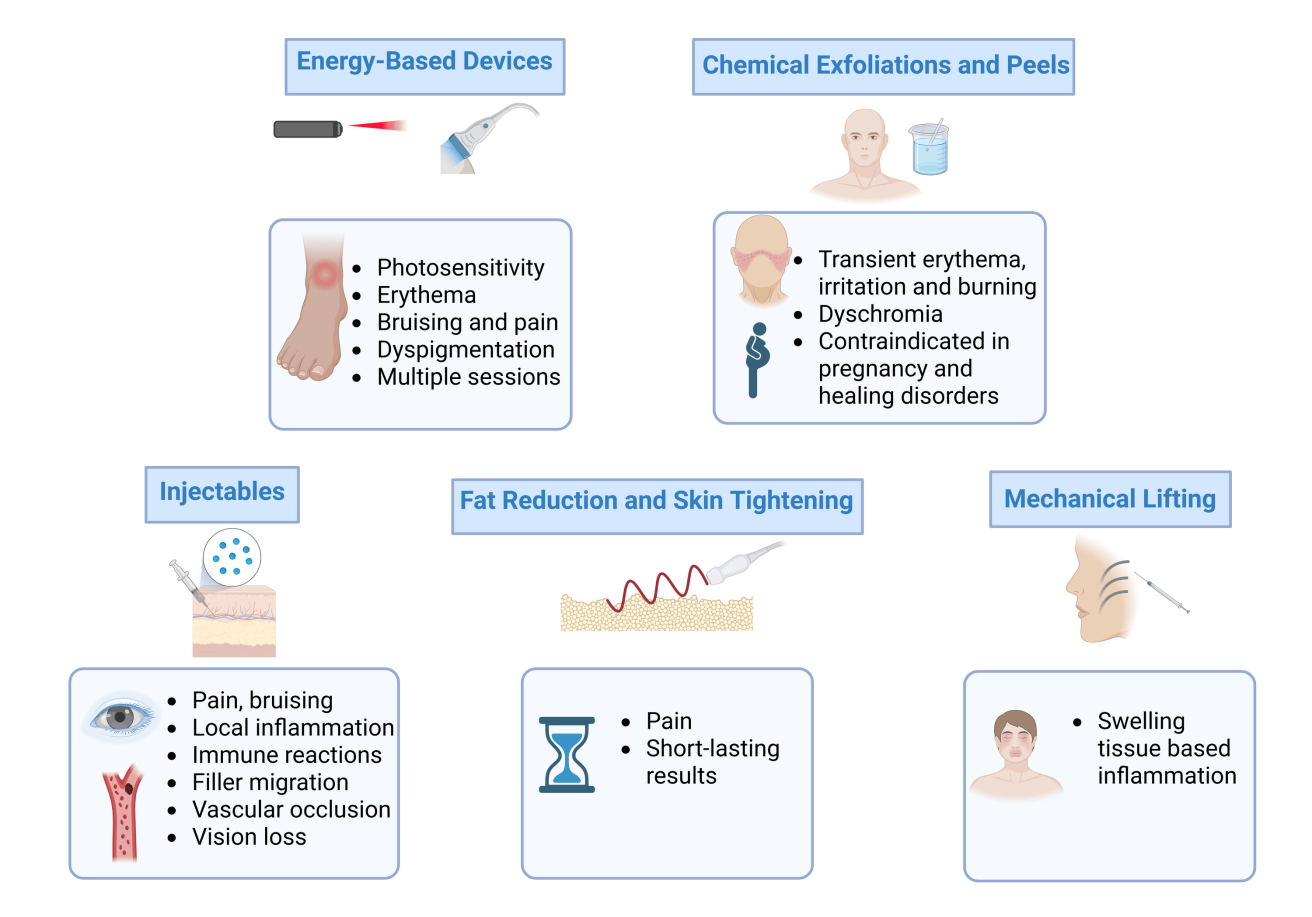
Common complications and limitations of non-surgical anti-aging techniques References [9,10,16,23,30,39,44] Image credits: Sergio Camilo-Torres; created in BioRender.com

When practicing aesthetic medicine, it is highly beneficial to recommend suitable treatments by classifying interventions according to the specific layers of the skin they target and their relevance to those anatomical structures (Figure 2) [60]. Equally important is assessing the patient’s expectations and understanding regarding their aesthetic needs. This process should also involve a comprehensive discussion of the proposed treatments' potential risks, adverse effects, and inherent limitations; we summarize common complications and limitations in Figure 3 [64].

## Conclusions

In conclusion, as the discipline of aesthetic medicine advances, this comprehensive review reinforces the increasing clinical significance of non-surgical and minimally invasive interventions in facial rejuvenation. The shift away from traditional surgical techniques toward less invasive modalities reflects a broader trend driven by patient demand for treatments that are safer, require minimal downtime, and offer natural-looking results.

However, these procedures are not without limitations. The review identifies several adverse effects, underscoring the importance of anatomical knowledge and patient-specific risk assessment in clinical practice. The evidence also highlights the necessity of combining treatments to achieve multi-layered rejuvenation.

Therefore, future research should focus on well-designed randomized controlled trials and longitudinal studies that not only assess efficacy but also evaluate long-term safety, patient satisfaction, and cost-effectiveness across diverse populations and skin types. These insights will be pivotal in refining evidence-based clinical guidelines, enhancing complication management strategies, and ensuring outcomes that are not only aesthetically pleasing but also ethically sound, sustainable, and aligned with patient expectations.

Future research should aim to refine clinical guidelines through well-structured comparative studies, and randomized controlled trials, ensuring that aesthetic outcomes are effective but also safe, sustainable, and aligned with patient expectations and needs, based on evidence-based science.
